# Long Non-coding RNA HOXA11-AS Facilitates Proliferation of Lung Adenocarcinoma Cells *via* Targeting the Let-7c-5p/IGF2BP1 Axis

**DOI:** 10.3389/fgene.2022.831397

**Published:** 2022-03-17

**Authors:** Xiaodong Lv, Zhixian Fang, Weibo Qi, Yufen Xu, Wenyu Chen

**Affiliations:** ^1^ Department of Respiration, Affiliated Hospital of Jiaxing University, Jiaxing, China; ^2^ Department of Cardiothoracic Surgery, Affiliated Hospital of Jiaxing University, Jiaxing, China; ^3^ Department of Oncology, Affiliated Hospital of Jiaxing University, Jiaxing, China

**Keywords:** HOXA11-AS, let-7c-5p, IGF2BP1, LUAD, proliferation

## Abstract

**Objective:** This study investigates the relationship between the HOXA11-AS/let-7c-5p/IGF2BP1 regulatory axis and lung adenocarcinoma.

**Methods:** The expression levels of HOXA11-AS, let-7c-5p, and IGF2BP1 were evaluated in LUAD tissue and cell lines. Subcellular fractionation detection assay was adopted to verify the HOXA11-AS distribution in LUAD cells. The interaction relationship between let-7c-5p and HOXA11-AS or IGF2BP1 was validated by dual-luciferase reporter detection. In RNA binding protein immunoprecipitation assay, the binding relationship between HOXA11-AS and let-7c-5p was identified. The cell viability of transfected cells was tested by the Cell Counting Kit-8 assay. The mouse xenograft model was used to identify the effect of HOXA11-AS on tumor growth *in vivo*.

**Results:** Upregulation of lncRNA HOXA11-AS was found in LUAD, and suppression of HOXA11-AS could suppress the proliferative ability of LUAD cells. The let-7c-5p was expressed to be downregulated, which played an inhibitory role in LUAD cell proliferation. Let-7c-5p was negatively regulated by HOXA11-AS. HOXA11-AS promoted LUAD cell proliferation, while let-7c-5p had an inverse effect. Besides, IGF2BP1, regulated by let-7c-5p, had a positive relation with HOXA11-AS, while overexpression of IGF2BP1 could suppress the inhibition of silencing HOXA11-AS on LUAD cell proliferation. Experiments on mice confirmed that HOXA11-AS facilitated LUAD cell growth *in vivo* through regulating the let-7c-5p/IGF2BP1 axis.

**Conclusion:** HOXA11-AS promoted LUAD cell proliferation by targeting let-7c-5p/IGF2BP1, which could be potential molecular targets for LUAD.

## Introduction

The incidence of lung cancer is increasing globally, with about 2.1 million new confirmed cases each year. It has become the leading cause of cancer deaths worldwide (Bray et al., 2018). Non‐small cell lung cancer (NSCLC) accounts for almost 80% of all lung cancer cases, of which lung adenocarcinoma (LUAD) is the most prevalent subtype (Martin and Leighl, 2017). Though treatments including surgical operation, chemotherapy, and molecular targeted therapy have been improved, prognosis of LUAD remains poor with a 5-year survival rate lower than 10% (Gelsomino et al., 2019). Accordingly, understanding the detailed mechanisms related to LUAD development is essential to getting an early and accurate diagnosis, thus deciding the best treatment for LUAD patients.

It is known that over 70% of human genomes are transcribable, but just 2% belong to protein-coding genes, and the others are noncoding RNAs (ncRNA). Long noncoding RNAs (lncRNAs) refer to transcripts containing over 200 nucleotides. Increasing evidence reveals that lncRNA regulates broad biological processes, which include cell apoptosis, invasion, migration, and proliferation (Chen et al., 2017; Zhu et al., 2017; Kong et al., 2019). LncRNA, as a competitive endogenous RNA (ceRNA), may affect post-translational regulation through communication with miRNA response elements to change the expression of downstream target genes (Poliseno et al., 2010; Wang et al., 2017). Previous studies demonstrated that ceRNA regulatory networks play an important role in cancer initiation and progression (Xu et al., 2019a; Liu et al., 2019).

HOXA11-AS is a highly conserved homeodomain situated in Homeobox (HOX) gene cluster (chromosome 7p15.2)^11^. It can bind with particular DNA areas and mediates gene transcription during the process of tumorigenesis and embryogenesis (Xue et al., 2018; Chaudhary, 2021). Disorders in gene expression of HOX are found in many kinds of human cancers like lung cancer (Calvo et al., 2000) and bladder cancer (Cantile et al., 2003). HOX genes are classified into four types (A, B, C, and D), which were located on four different chromosomes. At the terminal of 5ʹ region of the HOXA cluster, there are 3 lncRNAs (HOXA10-AS, HOXA11-AS, and HOXA transcript), among which HOXA11-AS was originally found in mouse embryonic cDNA library with a probe for sensing HOXA11 cDNA sequences (Chau et al., 2002). HOXA11-AS can serve as ceRNA for particular miRNAs in a variety of cancer types to modulate target gene expression (Xue et al., 2018). Overexpression of HOXA11-AS promotes proliferation and invasion of gastric cancer by sponging miR-1297 (Sun et al., 2016). A study indicated that HOXA11-AS of cervical cancer may perform a function *via* regulating the transcription of HOXA11 expression (Dear et al., 1993). For LUAD, HOXA11-AS is proved to be relevant to cisplatin resistance of LUAD cells (Zhang et al., 2019). Nevertheless, there are no detailed studies concerning the effect of HOXA11-AS on LUAD cell proliferation. Therefore, identifying the biological function of HOXA11-AS, combined with its downstream target genes, can provide a novel target for LUAD prognosis and treatment.

Taking HOXA11-AS as the research object, this study investigated the expression levels of HOXA11-AS, let-7c-5p, and IGF2BP1, and their targeted relationship in LUAD based on the principle of ceRNA. We also validated the impact of gene expression on LUAD progression *via in vitro* and *in vivo* assays. The HOXA11-AS/let-7c-5p/IGF2BP1 axis might provide a new molecular target for LUAD treatment.

## Materials and Methods

### Collection of Samples

Matched samples of tumor tissue and adjacent normal tissue (>2 cm away from tumor margin) of 30 LUAD patients from January 2020 to January 2021 were procured from The First Hospital of Jiaxing. Before the operation, all subjects had not received any chemotherapy or radiotherapy. All the samples were separately diagnosed by at least three experienced pathologists, and they were stored in liquid nitrogen immediately at −80°C prior to use. Approval of the Ethics Committee of The First Hospital of Jiaxing was obtained. The informed consent was signed by the subjects before these clinical samples were used for research.

### Bioinformatics Methods

Expression data of mRNA (containing 59 normal and 535 tumor samples) and miRNA (containing 46 normal and 521 tumor samples), along with clinical samples of LUAD, were accessed from The Cancer Genome Atlas (TCGA) database (https://portal.gdc.cancer.gov/). Differential expression of miRNA and mRNA between the two groups was analyzed using package “edgeR”. The research object lncRNA was ascertained with literature review. Prediction of the miRNA interacting with HOXA11-AS was done on the basis of starBase (http://starbase.sysu.edu.cn/). With further consultation on databases involving TargetScan (http://www.targetscan.org/vert_72/), mirDIP (http://ophid.utoronto.ca/mirDIP/index.jsp#r), miRDB (http://mirdb.org/), miRTarBase (http://mirtarbase.mbc.nctu.edu.tw/php/index.php), and starBase (http://starbase.sysu.edu.cn/), downstream target genes of that miRNA were predicted. Correlation of the target mRNA with clinical stage of LUAD was then analyzed.

### Cell Culture and Transfection

Human LUAD cell lines A549 (ATCC®CCL-185™), HCC827 (ATCC®CRL-2868™), H1568 (ATCC®CRL-5876™), and H1975 (ATCC®CRL-5908™), as well as human normal bronchial epithelial cell line HBE4-E6/E7 (ATCC®CRL-2078™) were all accessed from the American Type Culture Collection (ATCC, United States). All cells were cultivated in RPMI 1640 medium (Gibco, Carlsbad, CA, United States) added with 10% fetal bovine serum (FBS) (Gibco, Carlsbad, CA, United States) and then nurtured in 5% CO_2_ at 37°C.

As for cell transfection, Lipofectamine 2000 (Thermo Fisher Scientific, Inc.) was adopted to transfect sh-HOXA11-AS, oe-HOXA11-AS, let-7c-5p mimic, let-7c-5p inhibitor, oe-IGF2BP1, or corresponding negative controls (GenePharma, Shanghai) into A549 cells in line with the standard procedure. Afterwards, they were cultured in corresponding mediums.

### qRT-PCR

Trizol reagent (Invitrogen) was utilized for isolating total RNA as guided by the instruction. PrimeScript RT kit (Takara, Dalian, China) was employed to achieve reverse transcription. As directed, qRT-PCR was run in triplicate by utilizing SYBR Prime Script RT-PCR kit (Takara, Dalian, China), with GAPDH as an endogenous regulator of lncRNA and mRNA, and U6 as that of miRNA. The results were analyzed in 2^−ΔΔCt^. All the primers involved are listed in Table 1.

**TABLE 1:** Primer sequences in qRT-PCR.

Primer	Sequence (5′-3′)
HOXA11-AS	F: GAG​TGT​TGG​CCT​GTC​CTC​AA
	R: TTG​TGC​CCA​GTT​GCC​TGT​AT
let-7c-5p	F: GAG​GTA​GTA​GGT​TGT​ATG​GTT​G
	R: GCA​GGG​TCC​CGA​GGT​ATT​C
IGF2BP1	F: AAGGCACAAGGCAGGATT
	R: GCA​GCT​CAT​TGA​CGG​TTT​T
GAPDH	F: TGC​CAA​ATA​TGA​TGA​CAT​CAA​GAA
	R: GGA​GTG​GGT​GTC​GCT​GTT​G
U6	F: CTCGCTTCGGCAGCACA
	R: AAC​GCT​TCA​CGA​ATT​TGC​GT

### Subcellular Localization

NE-PER™ Nucleus and Cytoplasmic Extraction Reagents (ThermoFisher, 78833, United States) were employed to fractionate the nucleus and cytoplasm. Prior to the assay, adherent cells (1×10^7^) were collected and suspended in 500 μl of cytoplasmic extraction reagent I (CERI). Cold CERII was supplemented after the suspended cell precipitate was incubated for 10 min on ice. The mixture was then centrifuged at maximum speed for 5 min. After that, the supernatant was moved to a novel test tube and kept at −80°C for storage. Following addition of cold nuclear extraction reagents and centrifugation, nucleus extract was then obtained. Finally, RNA was isolated from the two parts and analyzed through qRT-PCR by setting GAPDH and U6 as controls for cytoplasm and nucleus separately.

### Western Blot Analysis

Lysis buffer was used to lyse the cells, and BCA protein assay kit (Thermo Fisher Scientific) tested the concentration of the protein. Then, the protein was isolated with 10% SDS-PAGE and moved to 0.22 mm PVDF membrane (Millipore, Billerica, MA, United States) for electrophoresis. Later on, the membrane was blocked with phosphate buffer saline involving 5% bovine serum albumin for 2 h at room temperature. Afterwards, it was incubated with primary antibodies with different dilutions at 4°C overnight. The primary antibodies were IGF2BP1 (abcam, Cambridge, United Kingdom), GAPDH (abcam, Cambridge, United Kingdom), and Ki67 (abcam, Cambridge, United Kingdom). Goat anti-rabbit IgG H&L (HRP) was the secondary antibody. The protein level in each sample was normalized with respect to GAPDH. Each assay involved 3 repeated wells of each sample and all of them were repeated at least three times.

### CCK-8 Assay

Each well of the 96-well plate (Corning Costar) included 2×10^3^ cells, which were then used for 48 h of transfection and incubation. After that, each well was supplemented with CCK-8 (10 μl; Dojindo Laboratories, Mashiki-machi, Kumamoto, Japan). Finally, cell viability was measured at 450 nm at 0, 24, 48, and 72 h after adding CCK-8 for 1.5 h.

### Dual-Luciferase Reporter Detection

HOXA11-AS (HOXA11-AS-MUT/WT) or IGF2BP1 (IGF2BP1-MUT/WT) was utilized to construct the plasmids containing firefly luciferase reporter genes. In a 24-well plate, each well was inoculated with 5×10^4^ cells and stored overnight. The next day, recombinant plasmids and empty plasmids encoding firefly luciferase reporter genes were transfected with Lipofectamine 2000. Renilla luciferase reporter gene pRLCMV (Promega, Madison, WI, United States) was co-transfected into the cells to serve as normal transfection control. After 48 h, dual-luciferase reporter gene detection system (Promega) measured luciferase viability of reporter gene in line with the instructions of the manufacturer.

### RIP Assay

EZ-Magna RIP kit (Millipore, Billerica, MA, United States) was used to carry out RIP analysis with the instruction of the manufacturer. In short, RIP lysis buffer was used to lyse cells of 70%–80% confluence, and then magnetic beads were used to couple with human anti-Ago2 antibody (Millipore) as well as homologous IgG control (Millipore) in the RIP buffer. Afterwards, RNase-free DNase I and Proteinase K were applied to remove the DNAs and proteins in RIP compounds. The enrichment of targeted RNA in obtained RNAs was tested *via* qRT-PCR.

### Nude Mouse Assay

Female athymic BALB/c nude mice (National Laboratory Animal Center, Beijing, China) aged 4 weeks were raised without specific pathogens and the experiment was operated in line with the procedures admitted by the Medical Experimental Animal Management Committee of Nanjing City. Stably transfected A549 cells with sh-HOXA11-AS or scrambled shRNA were acquired. Then, the left lower limb of the mice was subcutaneously injected with 1 × 10^7^ cells, and measurement of the tumor size was conducted every 4 days. After being injected for 4 weeks, the nude mice were killed and the tumors were removed for follow-up experiments. This study rigorously followed the nursing and operating guidance of laboratory animals from the US National Institutes of Health (NIH) and the program won the approval of the Ethics Committee for Laboratory Animals of Nanjing Medical University.

### Immunocytochemistry

The primary tumor was removed and processed with hematoxylin and eosin (HE) staining and immunohistochemical staining based on the instructions of the procedure. The tumor samples of the nude mice were paraffin-embedded, and fixed with 4% paraformaldehyde. Tissue sections were then cut into slices, dewaxed, and hydrated with graded alcohol. By incubation in 3% H_2_O_2_, endogenous peroxidase activity was blocked. Microwave thermal induction, as well as 0.01 M citrate buffer (pH 6.0), was used for antigen retrieval. Immunocytochemistry detection was adopted on tissue slices of 4 μm thickness, with primary rabbit antibodies IGF2BP1 (abcam, Cambridge, United Kingdom) and Ki67 (abcam, Cambridge, United Kingdom) added for overnight incubation at 4°C, and then secondary antibody IgG H&L (abcam, Cambridge, United Kingdom) was added. After being stained by diaminobenzidine, samples were separately assessed by two pathologists.

### Statistical Methods

GraphPad Prism 8 software (GraphPad Software, Inc., La Jolla, CA) was employed to process all of the data. All assays were repeated three times biologically and technically, with their outcomes represented as mean ± standard deviation. *T*-test was utilized to analyze the differentiation between two groups and one-way analysis of variance was performed for comparisons between multi groups. *p* < 0.05 indicated statistical significance.

## Results

### HOXA11-AS is Upregulated in Lung Adenocarcinoma With Implications on Lung Adenocarcinoma Cell Proliferation

**FIGURE 1:**
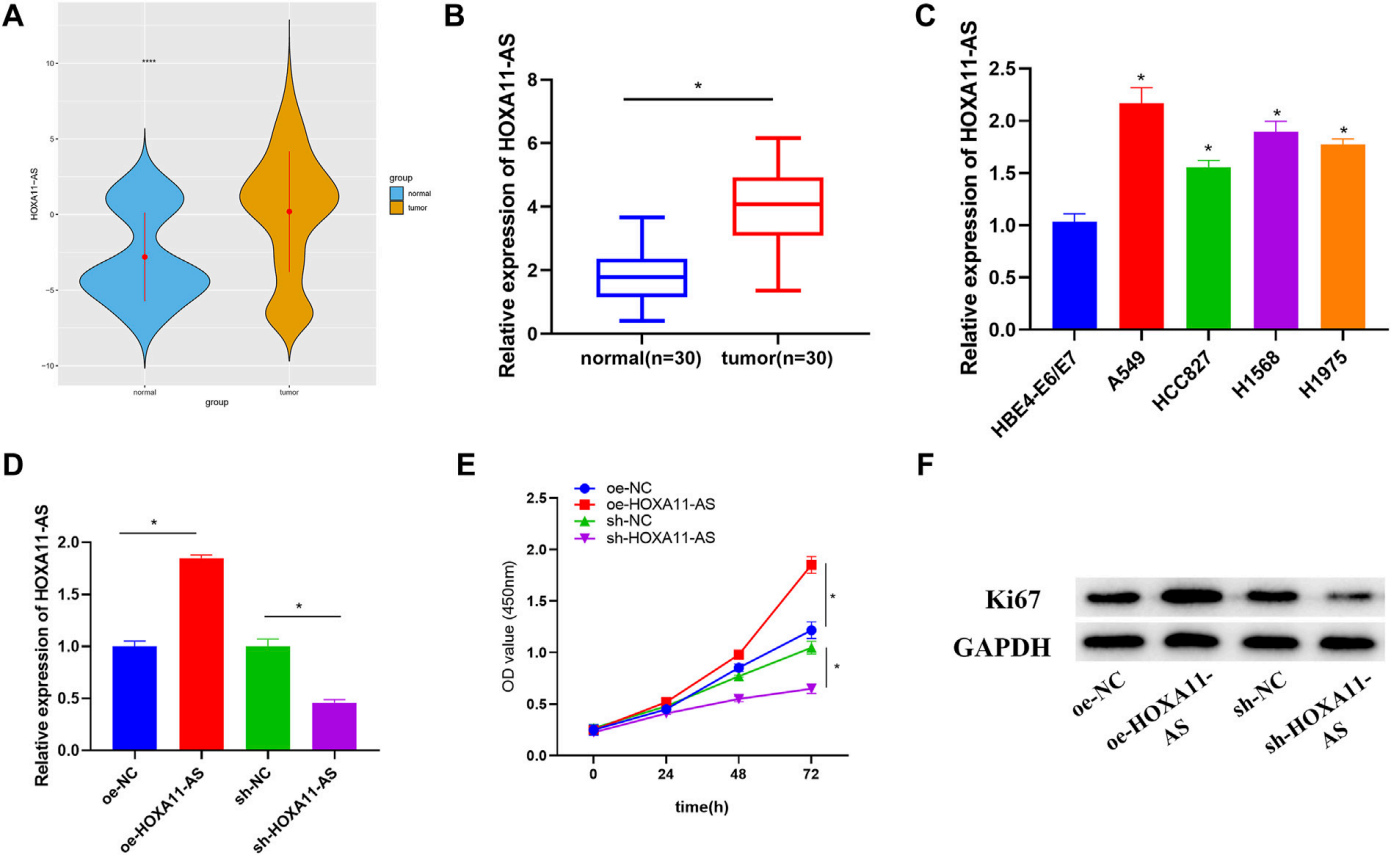
Upregulated HOXA11-AS in LUAD with implications on LUAD cell proliferation. **(A)** TCGA-LUAD dataset was employed to detect HOXA11-AS expression (blue represents normal tissue while orange represents tumor tissue). **(B)** HOXA11-AS expression was further measured in LUAD tissue (*n* = 30) and adjacent tissue (*n* = 30). **(C)** By qRT-PCR, HOXA11-AS expression in normal cell line and LUAD cell lines was presented. **(D)** qRT-PCR confirmed the transfection efficiency of HOXA11-AS. **(E)** Cell viability was observed through CCK-8. **(F)** Expression of cell proliferation-related protein Ki67 was exhibited *via* Western blot; *p < 0.05.

Expression data and clinical data of LUAD were accessed from TCGA database. Through observation, we found that HOXA11-AS was considerably elevated in LUAD tissue in comparison with that in normal lung tissue (Figure 1A). Besides, HOXA11-AS expression was also remarkably increased in clinical tumor samples (Figure 1B). Hence, the lncRNA HOXA11-AS was selected as the research object. Investigation on its expression in LUAD cell lines A549, HCC827, H1568, and H1975 and normal cell line HBE4-E6/E7 verified a remarkable elevation of HOXA11-AS expression in LUAD cell lines (Figure 1C). A549 was selected for subsequent experiments since it witnessed the most obvious expression variance. Then, oe-HOXA11-AS, sh-HOXA11-AS, as well as their paired negative control were transfected into A549 cell line, respectively. Further analysis could be conducted after the confirmation of successful transfection *via* qRT-PCR (Figure 1D). In CCK-8 assay, viability of cells in each group was evaluated. As presented in Figure 1E, cell viability dramatically increased when HOXA11-AS was overexpressed, whereas the viability significantly reduced in the experimental group with HOXA11-AS expression suppressed. Then, the expression of protein Ki67 related to proliferation was detected by Western blot. The result indicated a great rise of Ki67 expression in cells with overexpression of HOXA11-AS, and a distinct decrease in cells with inhibition of HOXA11-AS (Figure 1F). Collectively, overexpression of HOXA11-AS was identified to facilitate LUAD cell proliferation.

### HOXA11-AS Acts as a Sponge for Let-7c-5p

**FIGURE 2:**
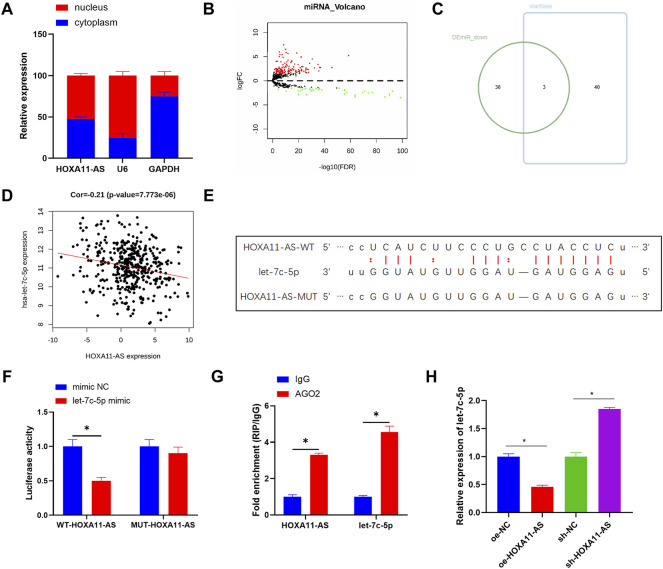
HOXA11-AS sponges let-7c-5p. **(A)** Subcellular fractionation evaluated the expression of HOXA11-AS in nucleus and cytoplasm. **(B)** Differential analysis on miRNA in two groups was performed and the result was shown in the Volcano Plot, with red indicating upregulated genes and green indicating downregulated genes. **(C)** Venn diagram depicted the intersection of predicted target miRNAs and differentially downregulated miRNAs. **(D)** The scatter plot described the correlation between HOXA11-AS and let-7c-5p. **(E)** The binding sites between HOXA11-AS and let-7c-5p were predicted on starBase database. **(F,G)** The target binding of let-7c-5p to HOXA11-AS was demonstrated in RIP assay and dual-luciferase reporter detection. **(H)** Let-7c-5p expression in each group of cells after transfection was determined in qRT-PCR assay; *p < 0.05.

Aiming to ascertain the biological function of HOXA11-AS in LUAD cells, subcellular fractionation was employed to describe the position of HOXA11-AS in cells. Results showed HOXA11-AS expression in both cell nucleus and cytoplasm (Figure 2A), which implied that HOXA11-AS might perform a function in cytoplasm. Hence, a bold conjecture was proposed that the regulation function of HOXA11-AS was achieved by its interaction with miRNA as a ceRNA in cytoplasm. A total of 186 differential miRNAs were observed through bioinformatics analysis underlying the expression data of miRNA in the TCGA-LUAD dataset (Figure 2B). Based on the recognized negative regulatory relationship between lncRNA and miRNA in the ceRNA hypothesis, the 39 downregulated miRNAs obtained by differential analysis were intersected with the predicted results for HOXA11-AS on the starBase database. Finally, 3 DEmiRNAs were found to have binding sites with HOXA11-AS (Figure 2C). Among them, let-7c-5p and HOXA11-AS were significantly and negatively correlated (Figure 2D). In accordance with the putative binding site between HOXA11-AS and let-7c-5p (Figure 2E), we launched dual-luciferase reporter detection, thus establishing the targeting relationship between HOXA11-AS and let-7c-5p (Figure 2F). RIP assay displayed that after the addition of AGO2 antibody, HOXA11-AS and let-7c-5p levels in the precipitate were upregulated, which also confirmed their binding relationship (Figure 2G). Besides, let-7c-5p expression was dramatically decreased in HOXA11-AS overexpressed cells, whereas its expression was significantly increased in HOXA11-AS suppressed cells through qRT-PCR detection (Figure 2H). That also supported the claim that let-7c-5p and HOXA11-AS had a negative regulatory relationship.

### Let-7c-5p is Poorly Expressed in Lung Adenocarcinoma Cells, Suppresses Lung Adenocarcinoma Cell Proliferation, and can Alleviate the Promotion of HOXA11-AS on Lung Adenocarcinoma Cell Proliferation

**FIGURE 3:**
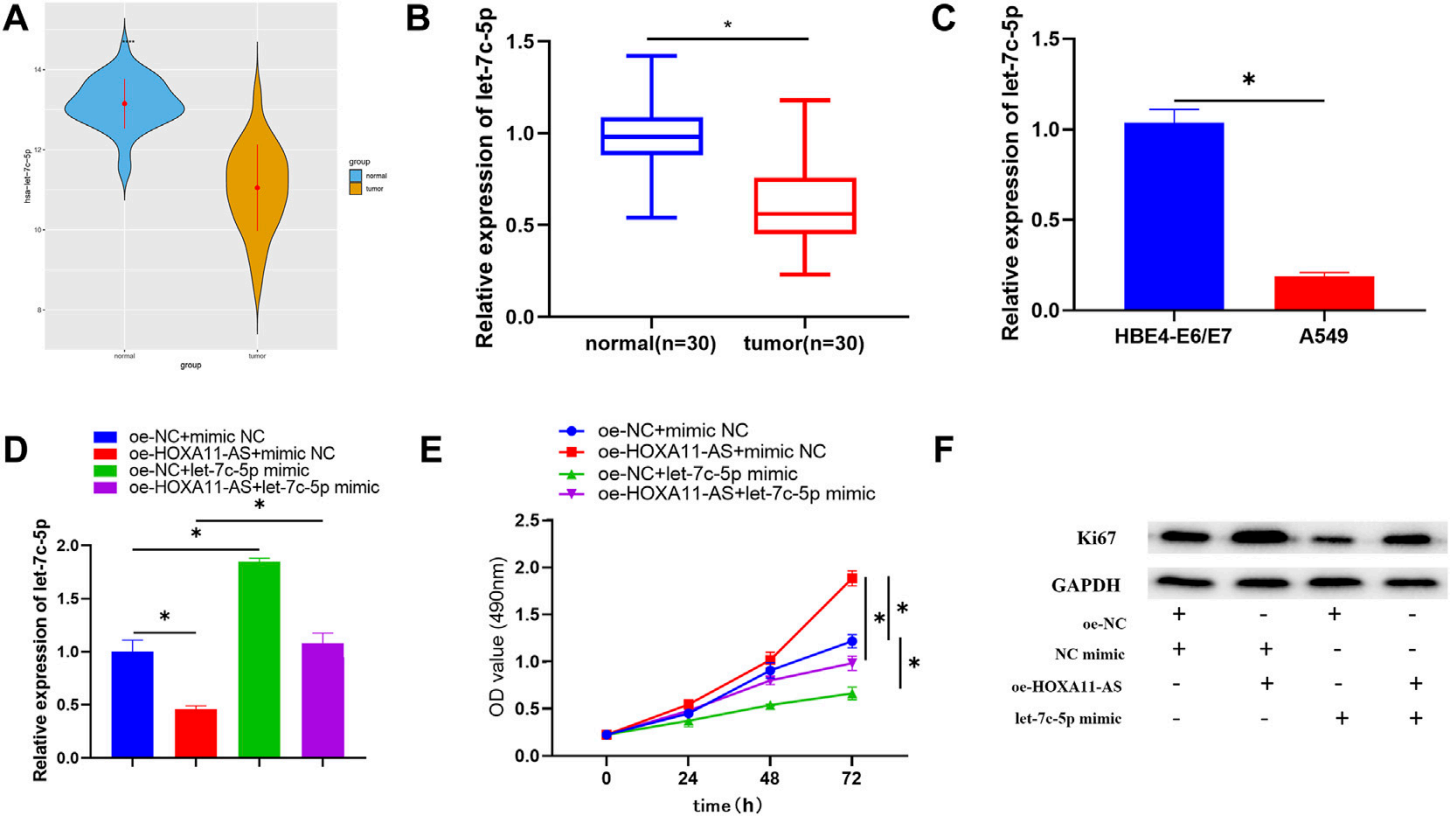
Let-7c-5p is poorly expressed in LUAD cells, hinders LUAD cell proliferation, and can alleviate the capacity of HOXA11-AS on proliferating the LUAD cells. **(A,B)** Let-7c-5p expression in LUAD tissue and adjacent normal tissue. **(C)** Expression of let-7c-5p in normal cell line HBE4-E6/E7 and LUAD cell lines A549 evaluated in qRT-PCR assay. **(D)** let-7c-5p expression in each transfection group measured by performing qRT-PCR. **(E)** CCK-8 was applied to test the viability of cells. **(F)** Ki67 expression was measured by Western blot; *p < 0.05.

Based on the aforementioned study, the negative regulation between let-7c-5p and HOXA11-AS was clarified. TCGA-LUAD data exhibited remarkable downregulation of let-7c-5p in LUAD tissue (Figure 3A). The reduction of let-7c-5p was also observed in LUAD tissue through qRT-PCR (Figure 3B). By adopting qRT-PCR again, let-7c-5p expression in LUAD cell lines and HBE4-E6/E7 cell line was detected and the result showed a considerable decrease in let-7c-5p expression in LUAD cell lines (Figure 3C). On the basis of this, we designed rescue experiments to validate whether the HOXA11-AS/let-7c-5p axis could influence cell proliferation. Figure 3D displayed the let-7c-5p expression in each group of cells after transfection. Detection results for cell viability and the expression of Ki67 by adopting CCK-8 and Western blot manifested that the cell proliferative ability was remarkably suppressed in the group of single let-7c-5p overexpression. In the group of single HOXA11-AS overexpression, cell proliferation was dramatically enhanced. However, such effects could be attenuated when let-7c-5p and HOXA11-AS were simultaneously overexpressed (Figures 3E,F). Accordingly, we verified that let-7c-5p could hinder the proliferation of LUAD cells and mitigate the ability of HOXA11-AS on promoting proliferation of LUAD cells.

### IGF2BP1 Serves as a Downstream Target Gene Modulated by Let-7c-5p

**FIGURE 4:**
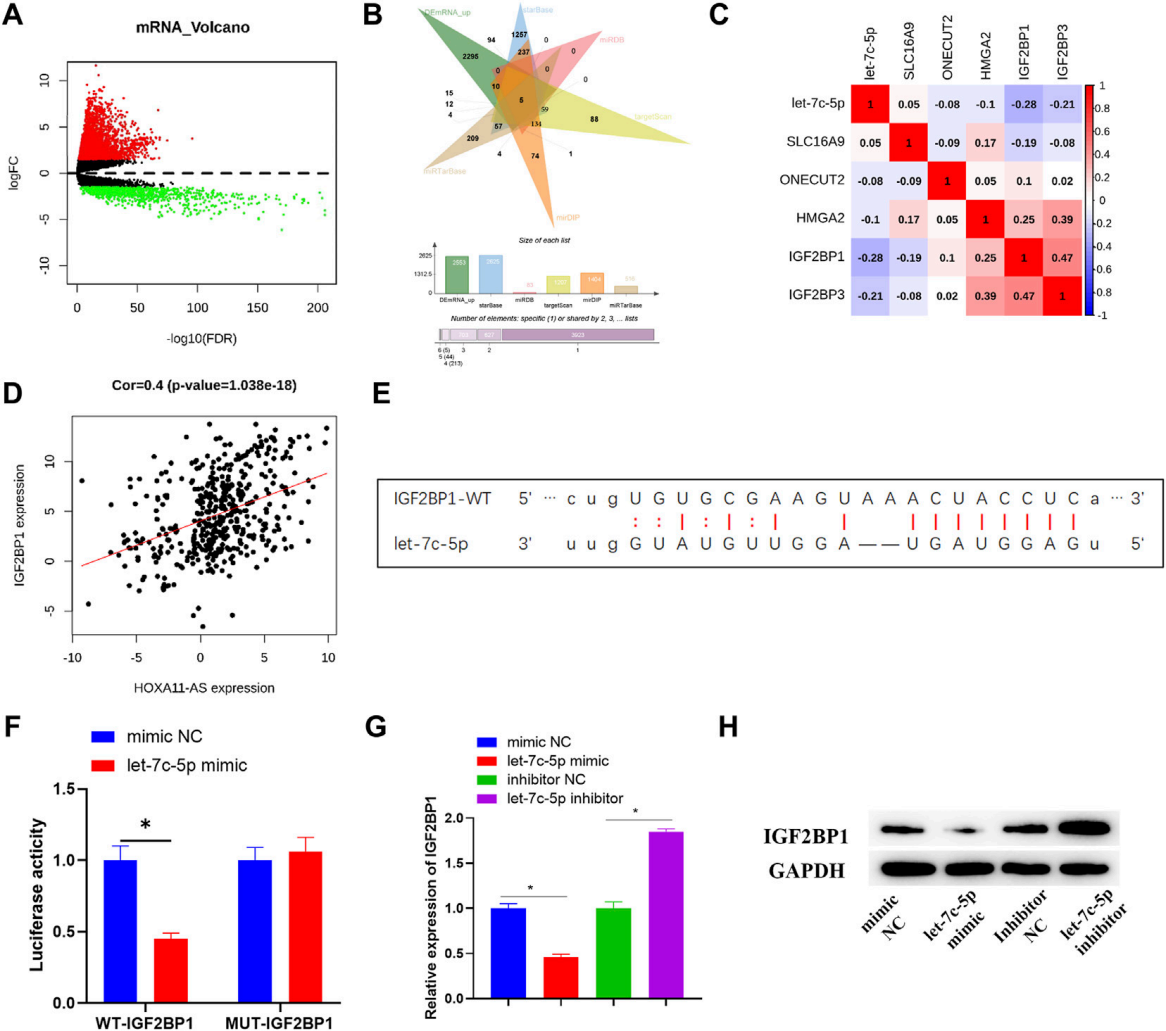
IGF2BP1 is targeted and regulated by let-7c-5p. **(A)** Differentially expressed mRNAs in normal group and tumor group was analyzed and the result was displayed in the Volcano Plot, in which red represents upregulated mRNAs and green represents downregulated mRNAs. **(B)** Venn diagram depicted the intersection of predicted target mRNAs and differentially upregulated mRNAs. **(C,D)** Pearson correlation analysis on potential target mRNAs and let-7c-5p, and the correlation analysis between IGF2BP1 and HOXA11-AS. **(E)** The target binding sequence of let-7c-5p on IGF2BP1 3′UTR was predicted on starBase database. **(F)** The target binding relationship of let-7c-5p and IGF2BP1 identified by dual-luciferase reporter detection. **(G)** qRT-PCR assessed expression level of IGF2BP1 mRNA after transfection in each group. **(H)** The expression of IGF2BP1 protein in each group of cells was tested in Western blot assay; *p < 0.05.

In order to consummate understanding of the regulatory mechanism of HOXA11-AS, a further study was carried out on the downstream target gene of let-7c-5p. In total, 3,591 differential mRNAs were acquired by differentially analyzing TCGA-LUAD dataset with the “edgeR” package (Figure 4A). Then, 5 potential downstream target genes were discovered after the intersection between the upregulated mRNAs accessed from differential analysis and the predicted results, based on the principle of ceRNA (Figure 4B). Among that, IGF2BP1 exhibited a negative and the strongest correlation with let-7c-5p (Figure 4C), and a significant positive correlation with HOXA11-AS (Figure 4D). Hence, it was selected for further studies. According to the targeting binding sequence between IGF2BP1 and let-7c-5p (Figure 4E), their targeting binding relationship was proved *via* dual-luciferase reporter assay (Figure 4F). In addition, both the outcomes of Western blot and qRT-PCR indicated a notable downregulation of IGF2BP1 in let-7c-5p overexpressed cells, while the expression of IGF2BP1 was considerably increased with the suppression of let-7c-5p (Figures 4G,H). That verified a negative regulatory relationship between IGF2BP1 and let-7c-5p.

### IGF2BP1 is Upregulated in Lung Adenocarcinoma Cells, and the Inhibition of IGF2BP1 can Mitigate the Promotion of Overexpressed HOXA11-AS on Lung Adenocarcinoma Cell Proliferation

**FIGURE 5:**
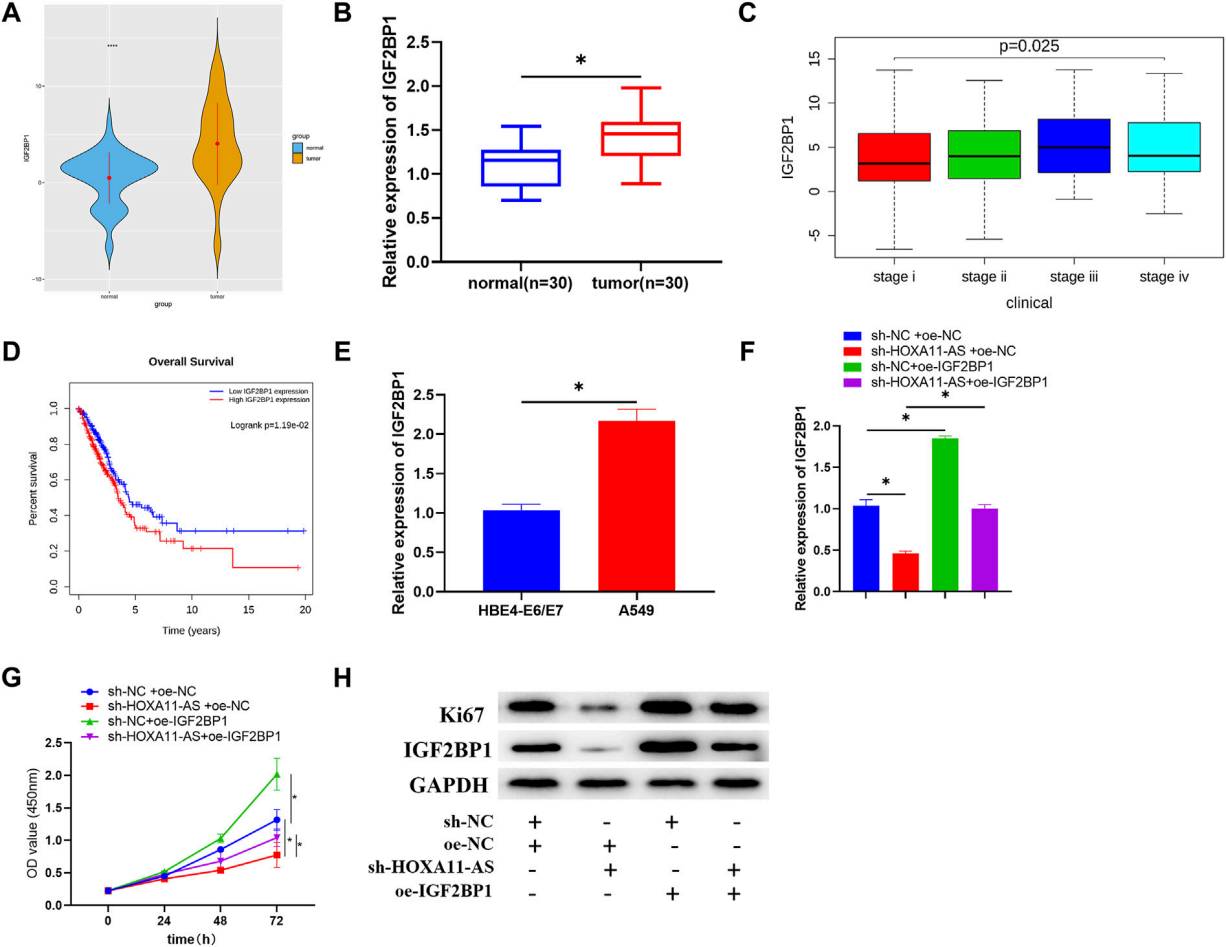
IGF2BP1 is upregulated in LUAD cells, and the inhibition of IGF2BP1 can mitigate the promotion of overexpressed HOXA11-AS on LUAD cell proliferation. **(A)** Expression of target gene IGF2BP1 in normal and tumor group was depicted in boxplot (blue refers to normal tissue, orange refers to tumor tissue). **(B)** Expression of IGF2BP1 in tumor tissue (*n* = 30) and adjacent normal tissue (*n* = 30). **(C)** Expression of IGF2BP1 in various stages of LUAD (stage i: *n* = 279; stage ii: *n* = 124; stage iii: *n* = 85; stage iv: *n* = 26). **(D)** Relationship between IGF2BP1 and survival rate was estimated with red for high IGF2BP1 expression and blue for low IGF2BP1 expression. **(E)** qRT-PCR was conducted for the assessment of IGF2BP1 mRNA in normal cell line HBE4-E6/E7 and LUAD cell line A549. **(F)** IGF2BP1 mRNA in transfected cells was evaluated in qRT-PCR assay. **(G)** By carrying out CCK-8 assay, cell viability in each group was estimated. **(H)** Through Western blot, the expression of proliferation of Ki67 as well as IGF2BP1 in each group of cells was tested; *p < 0.05.

As shown in the TCGA-LUAD dataset, there was a significantly high expression level of IGF2BP1 in LUAD tissue (Figure 5A). Among the clinical samples we collected, IGF2BP1 expression was also dramatically high in tumor tissue (Figure 5B). Meanwhile, analysis of IGF2BP1 for correlation with clinical stage based on TCGA-LUAD dataset identified that expression of IGF2BP1 was positively correlated with clinical stages with considerably altered expression in different stages (Figure 5C), and the survival rate of patients with high IGF2BP1 expression was dramatically low, in comparison with those patients with low IGF2BP1 expression (Figure 5D). From the aspects of cell lines, IGF2BP1 expression was upregulated in LUAD cell lines relative to that in the HBE4-E6/E7 cell line (Figure 5E). A series of rescue experiments were then designed for further identification of the biological function of IGF2BP1 in LUAD cells, and the relationship between IGF2BP1 and HOXA11-AS. Results demonstrated that IGF2BP1 was remarkably downregulated and cell proliferation was correspondingly depressed, with single inhibition of HOXA11-AS, while overexpression of IGF2BP1 alone could facilitate cell proliferation. Notably, in the experimental group with simultaneous inhibition of HOXA11-AS and overexpression of IGF2BP1, IGF2BP1 was greatly upregulated and cell proliferation was largely elevated, in comparison with the group with single inhibition of HOXA11-AS (Figures 5F–H).

### HOXA11-AS Facilitates Lung Adenocarcinoma Cell Growth *in vivo via* Mediating the Let-7c-5p/IGF2BP1 Axis in Mice

**FIGURE 6:**
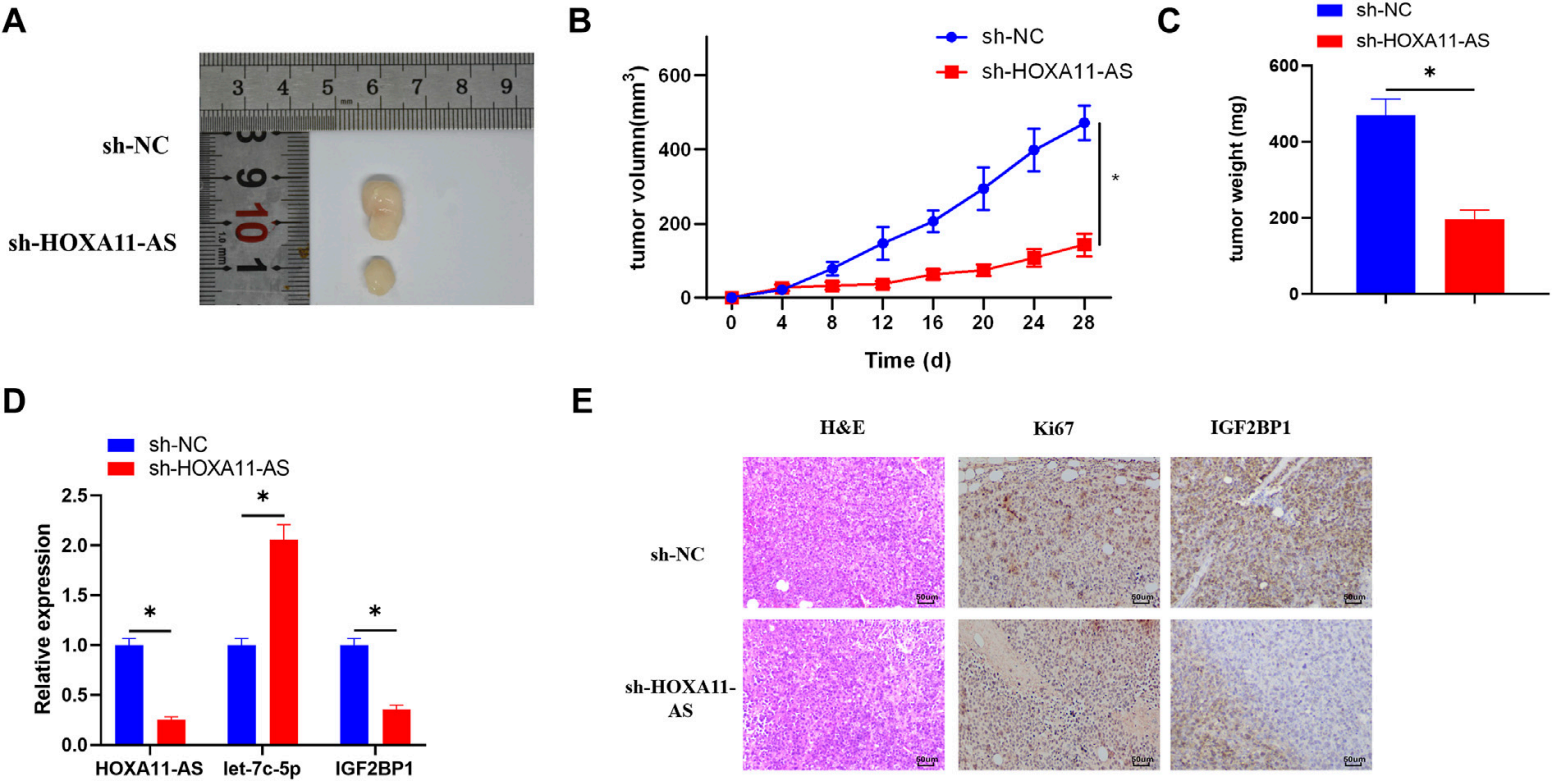
HOXA11-AS facilitates LUAD cell growth *in vivo via* mediating the let-7c-5p/IGF2BP1 axis in mice. **(A)** Tumor condition in the HOXA11-AS inhibited group and the control group. **(B)** Growth curve of the tumors in mice. **(C)** Weight of the tumors was recorded. **(D)** qRT-PCR assay was conducted to assess the expression of HOXA11-AS, let-7c-5p, and IGF2BP1 in tumor tissue of mice. **(E)** By performing immunohistochemical analysis, expression of Ki67 and IGF2BP1 was determined (×400); *p < 0.05.

In order to verify whether HOXA11-AS performs the same function *in vivo* and *in vitro*, the mice were respectively inoculated with cell lines that stably inhibited HOXA11-AS or the blank control cell lines. After 28 days of injection, the weight and size of the tumors in the HOXA11-AS suppressed group were observably smaller than those in the control group (Figures 6A–C). Besides, the HOXA11-AS inhibited group had relatively lower HOXA11-AS and IGF2BP1 expressions, but higher let-7c-5p expression, in comparison to those of the control group (Figure 6D). HE staining was performed on the tissue sections, and the results revealed that the proliferative ability of the experimental group that inhibited HOXA11-AS was far weaker than that of the control group. Immunohistochemical analysis of IGF2BP1 and proliferation-related protein Ki67 in the tissue sections reported that IGF2BP1 and Ki67 expression levels in the HOXA11-AS suppressed group were greatly low, in comparison with those in the control group (Figure 6E), which was consistent with the outcomes of cell experiments *in vitro*.

## Discussion

For many years, people conceived lncRNA as a “transcriptional noise” regardless of its influences on cells. However, lncRNA attracts more attention as a crucial regulator in biological processes. Multiple studies proved that lncRNA can regulate the process of tumor initiation and progression *via* its promotive or suppressive function (She et al., 2016; Liu et al., 2018; Lu et al., 2019). In this study, lncRNA HOXA11-AS was screened out from the TCGA-LUAD dataset. Our data identified that upregulation of HOXA11-AS significantly accelerated the proliferation of LUAD cell line A549, proposing an oncogenic role. A previous study indicated that HOXA11-AS promoted proliferation of non-small cell lung cancer cells through targeting miR-124 (Yu et al., 2017). Moreover, in the nude mouse model, suppression of HOXA11-AS could considerably inhibit tumor growth, suggesting the potential role of HOXA11-AS in the progression of LUAD, which is identical with previous research results about other cancers. For example, by sponging miR-146b-5p, HOXA11-AS can upregulate MMP16 to accelerate the development and metastasis of renal cancer cells (Yang et al., 2018). Besides, HOXA11-AS serves as a promotor in different types of cancer such as liver cancer (Zhan et al., 2018), gastric cancer (Liu et al., 2017), and glioma (Xu et al., 2019b). Nevertheless, in the field of LUAD, current research has so far touched upon on the relationship between HOXA11-AS and cisplatin resistance (Zhao et al., 2018). The current knowledge about the potential role of HOXA11-AS in the process of LUAD initiation and progress is still limited, which needs further explanation.

Thereafter, the most related miRNA let-7c-5p was screened out using bioinformatics methods so as to figure out the regulatory mechanism of HOXA11-AS in the LUAD cell line. Let-7 miRNA family was involved in modulation of oncogenic pathways, such as Myc and Ras, to function as a tumor suppressor in different types of cancer (Zhu et al., 2015; Nwaeburu et al., 2016). Reports claimed that let-7c-5p suppresses the proliferative ability of breast cancer cells *via* targeting ERCC6 and induces autophagy (Fu et al., 2017); let-7c-5p restrains cancer development by inhibiting c-Myc in liver cancer (Chen et al., 2019). It can serve as a prognostic factor of many cancers, such as LUAD (Yu et al., 2020), colorectal cancer (Zhou et al., 2018), malignant pleural mesothelioma (De Santi et al., 2017), and endometrial cancer (Xiong et al., 2014). Nevertheless, its function on the occurrence and development of LUAD has not yet been confirmed. We found that let-7c-5p was poorly expressed in both LUAD tumor tissues and cell lines through our study. It was negatively regulated by HOXA11-AS in cell lines *in vitro*, suggesting that HOXA11-AS could act as a ceRNA of let-7c-5p. Overexpression of let-7c-5p was identified to reduce the proliferation of LUAD cells, while rescue experiments demonstrated that compared to the experimental group of overexpressing HOXA11-AS alone, the group of overexpressing let-7c-5p and HOXA11-AS simultaneously had prominently recovered proliferative ability of LUAD cells. These results indicate that HOXA11-AS regulates LUAD progression *via* regulating let-7c-5p.

Downstream target gene IGF2BP1 of let-7c-5p is proved to be widely expressed in embryos and tumor tissues, to play an important role in controlling embryonic development (Pillas et al., 2010), and to serve as a carcinogen in most cancers including liver cancer (Jiang et al., 2017), glioblastomas (Wang et al., 2015), and gastric cancer (Gu et al., 2015). In this research, IGF2BP1 was actively expressed in both LUAD clinical tumor tissues and cell lines. Besides, overexpression of IGF2BP1 in LUAD cell lines *in vitro* notably accelerated cell proliferation. We also ascertained the target binding relationship between IGF2BP1 and let-7c-5p. According to the rescue experiment for the confirmation of the connection between IGF2BP1 and HOXA11-AS, overexpressed IGF2BP1 could alleviate the inhibition on LUAD cell proliferation caused by suppression of HOXA11-AS, which explained the positive regulatory role of HOXA11-AS towards IGF2BP1.

In sum, our data demonstrated a promising oncogenic role of HOXA11-AS in LUAD. HOXA11-AS facilitates LUAD cell proliferation *via* regulating the let-7c-5p*/*IGF2BP1 axis. Hence, HOXA11-AS is hoped to be a clinical target for treatment of LUAD.

## Data Availability

The datasets presented in this study can be found in online repositories. The names of the repository/repositories and accession number(s) can be found in the article/Supplementary Material.
