# Supplementary figures and images for: Long Non-coding RNA HOXA11-AS Facilitates Proliferation of Lung Adenocarcinoma Cells via Targeting the Let-7c-5p/IGF2BP1 Axis
Source: Front Genet. 2022 Mar 17;13:831397. doi: 10.3389/fgene.2022.831397 (PMC8969016; doi:10.3389/fgene.2022.831397)

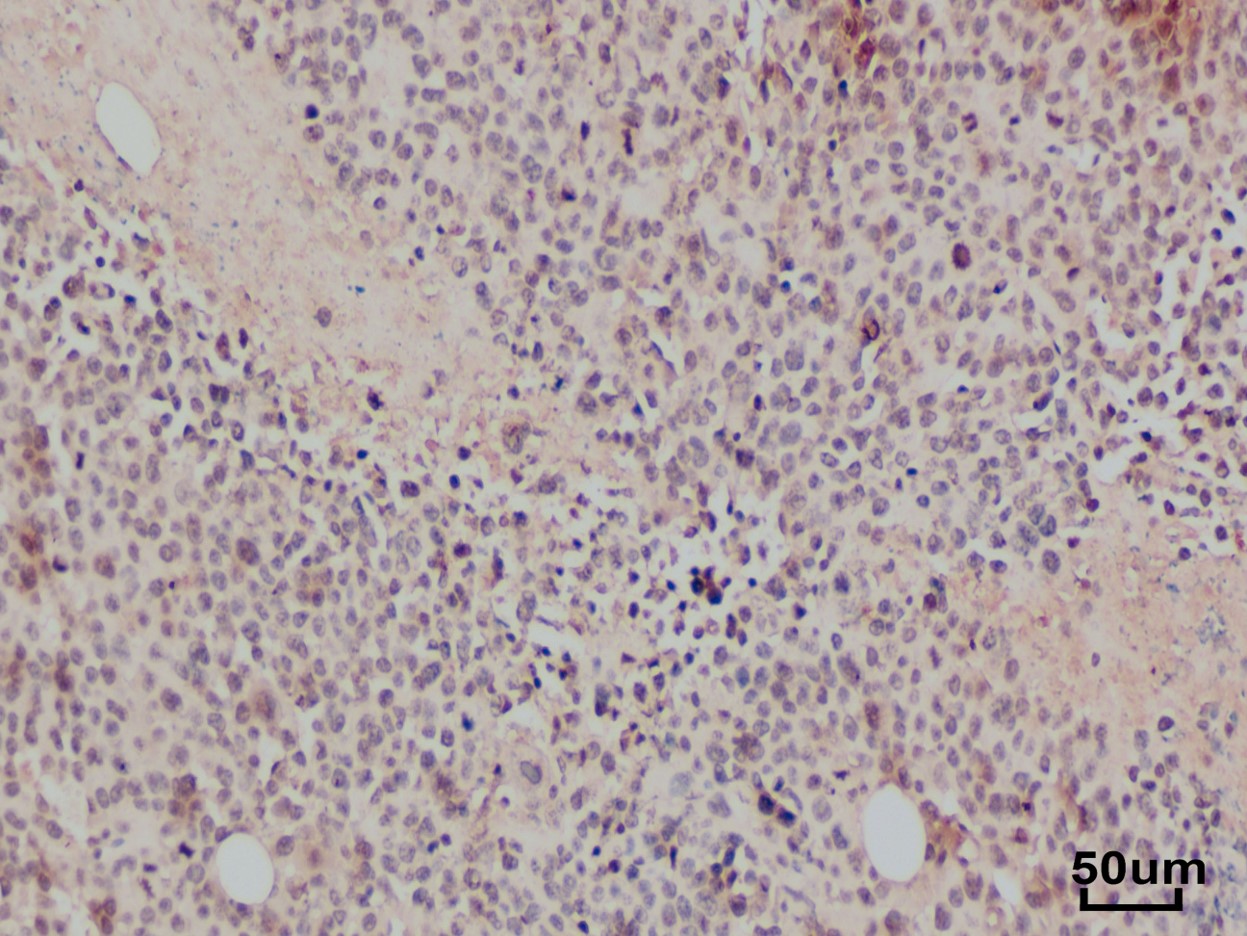

Supplement: Supplementary file 1 [file Image9.JPEG]

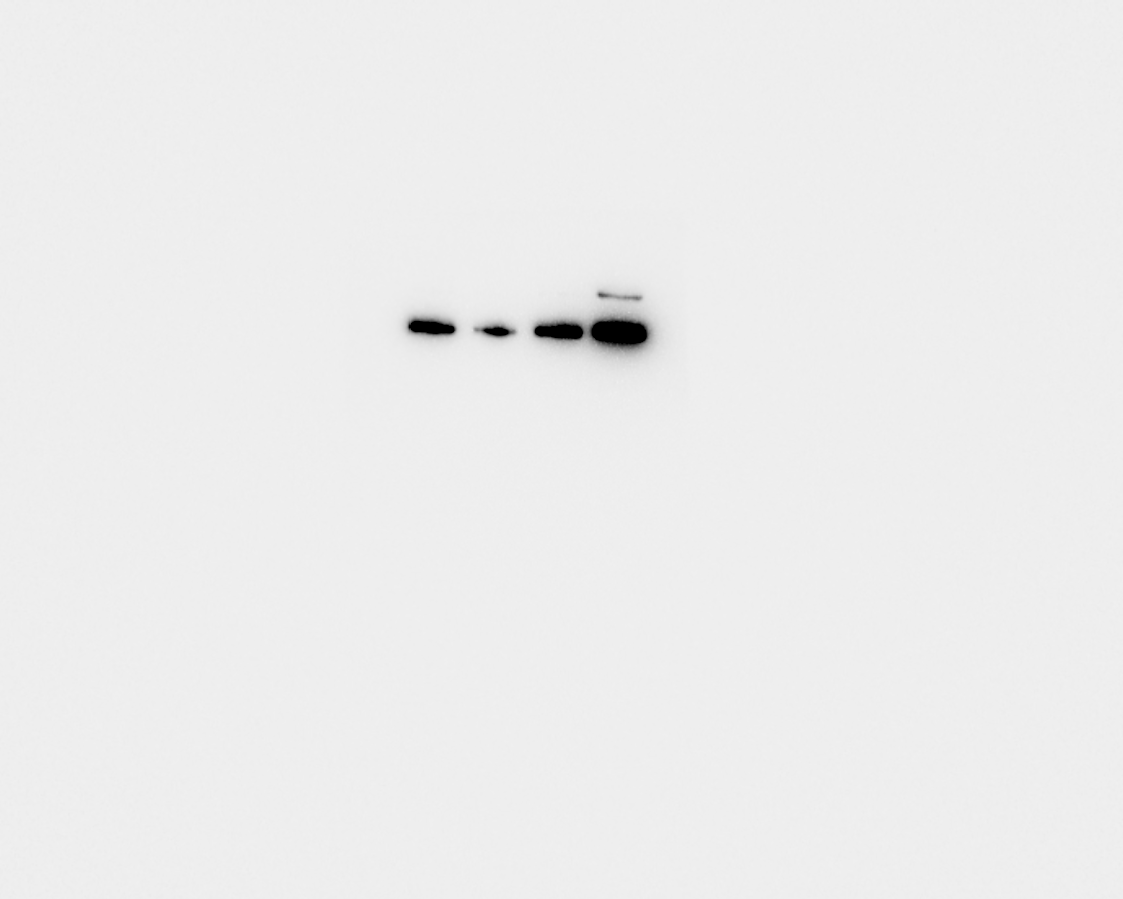

Supplement: Supplementary file 2 [file Image3.TIF]

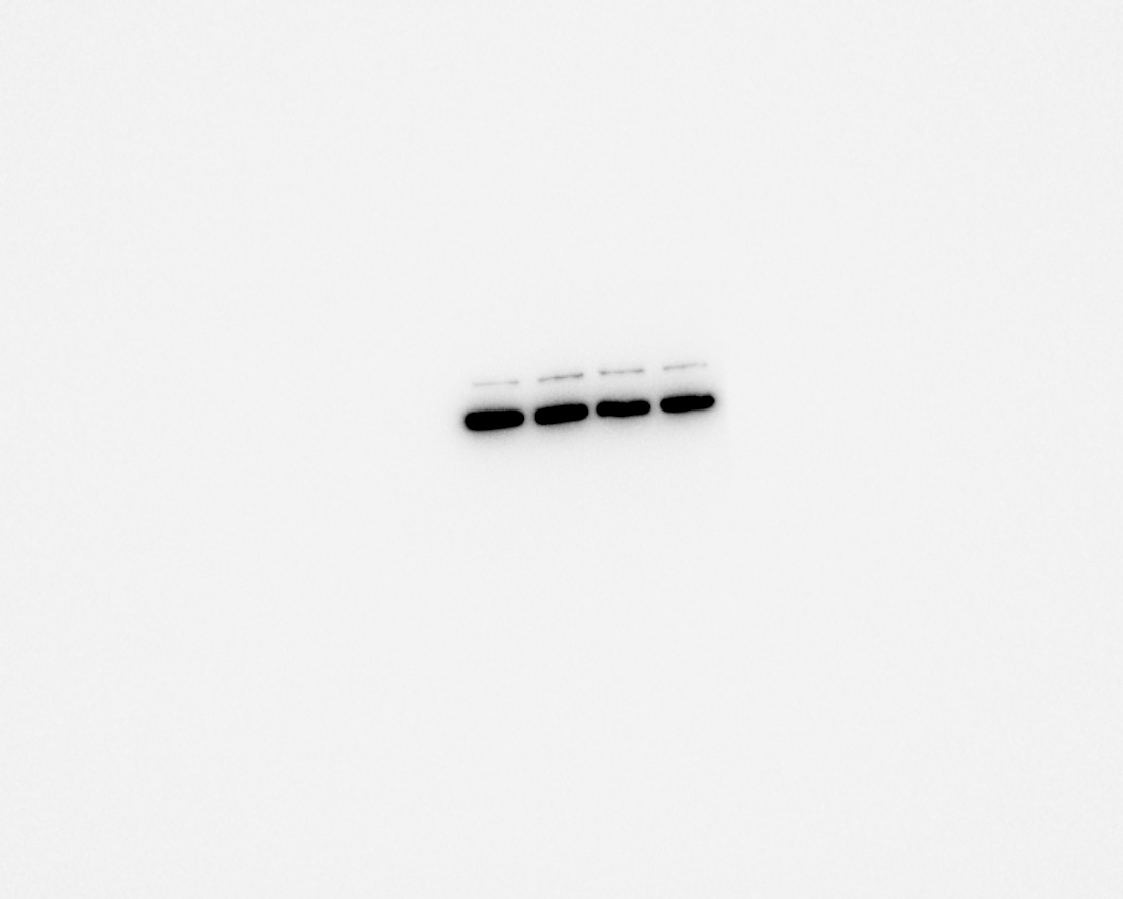

Supplement: Supplementary file 3 [file Image4.TIF]

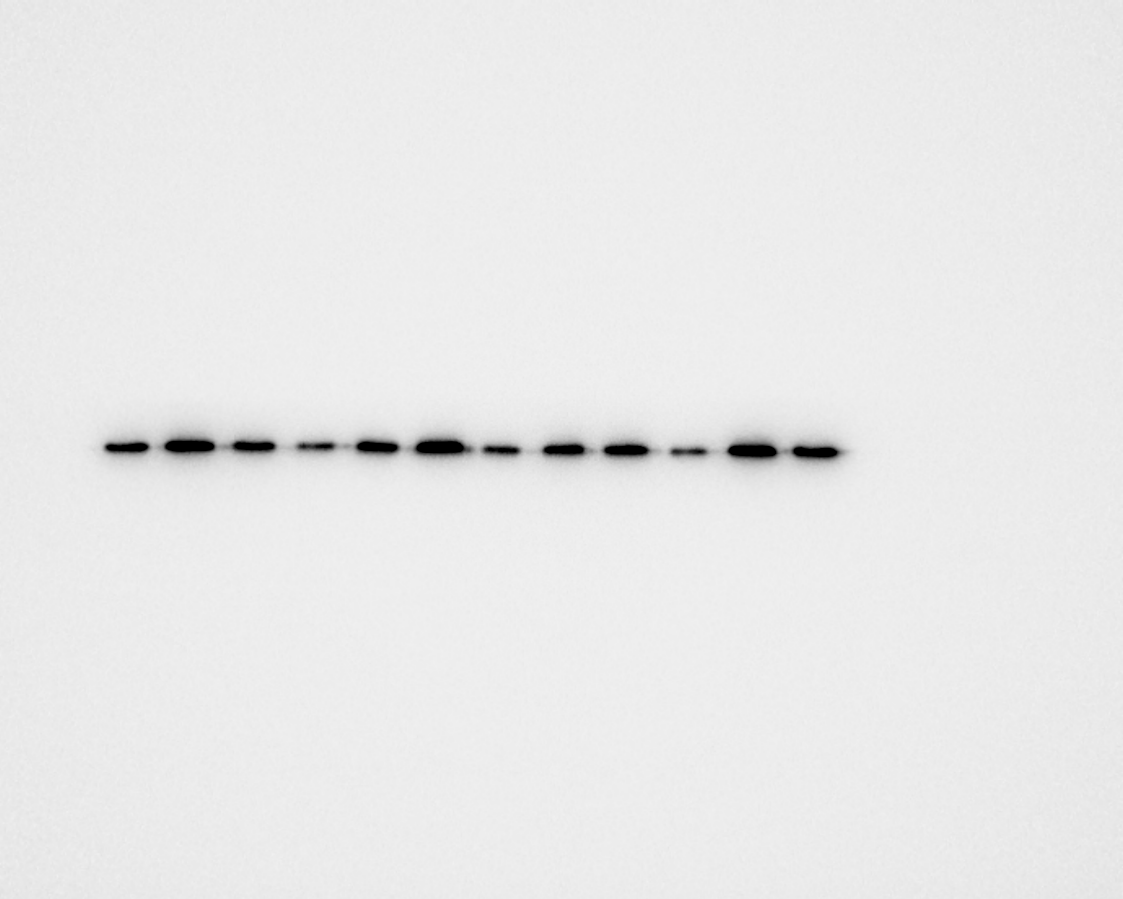

Supplement: Supplementary file 4 [file Image2.TIF]

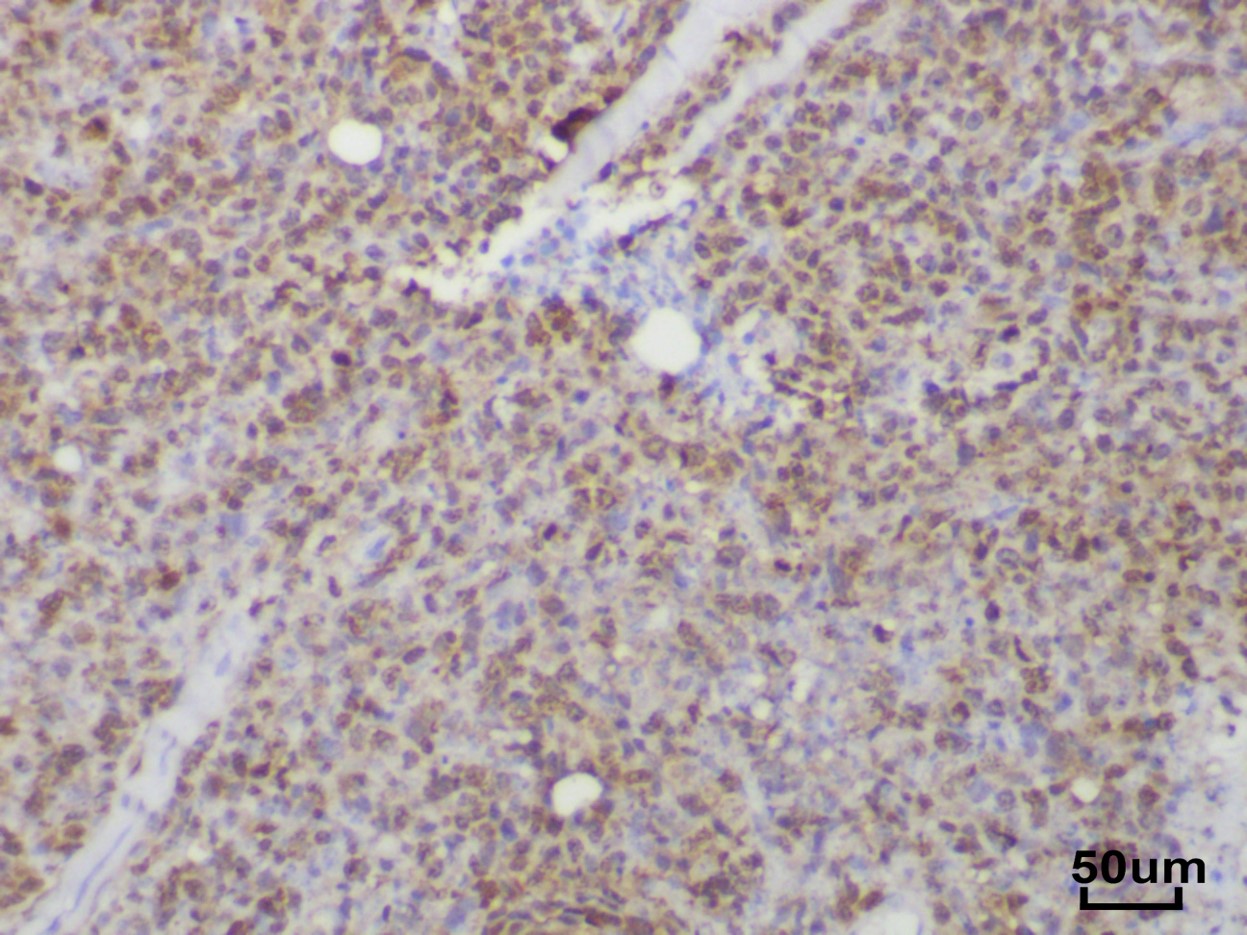

Supplement: Supplementary file 5 [file Image7.JPEG]

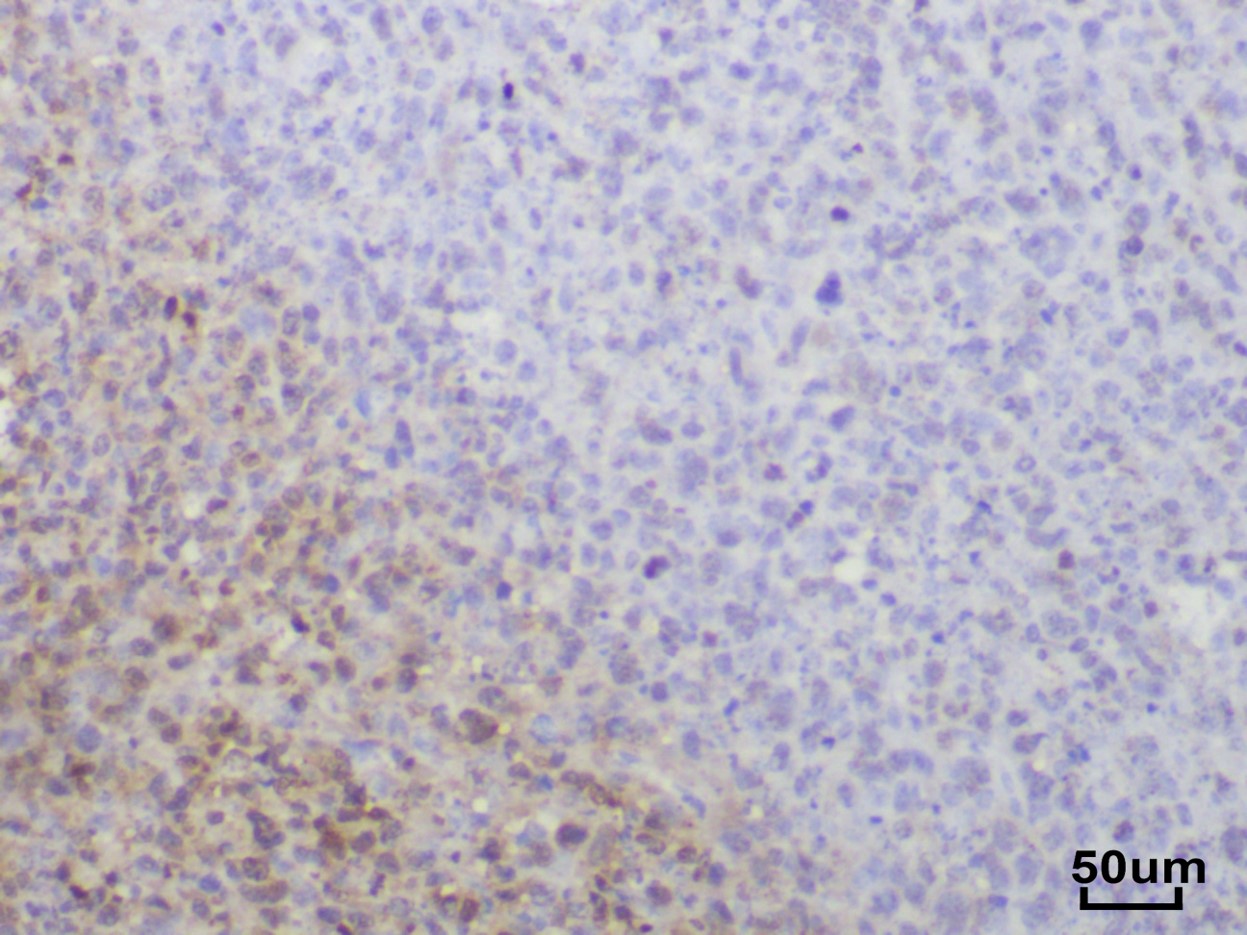

Supplement: Supplementary file 6 [file Image10.JPEG]

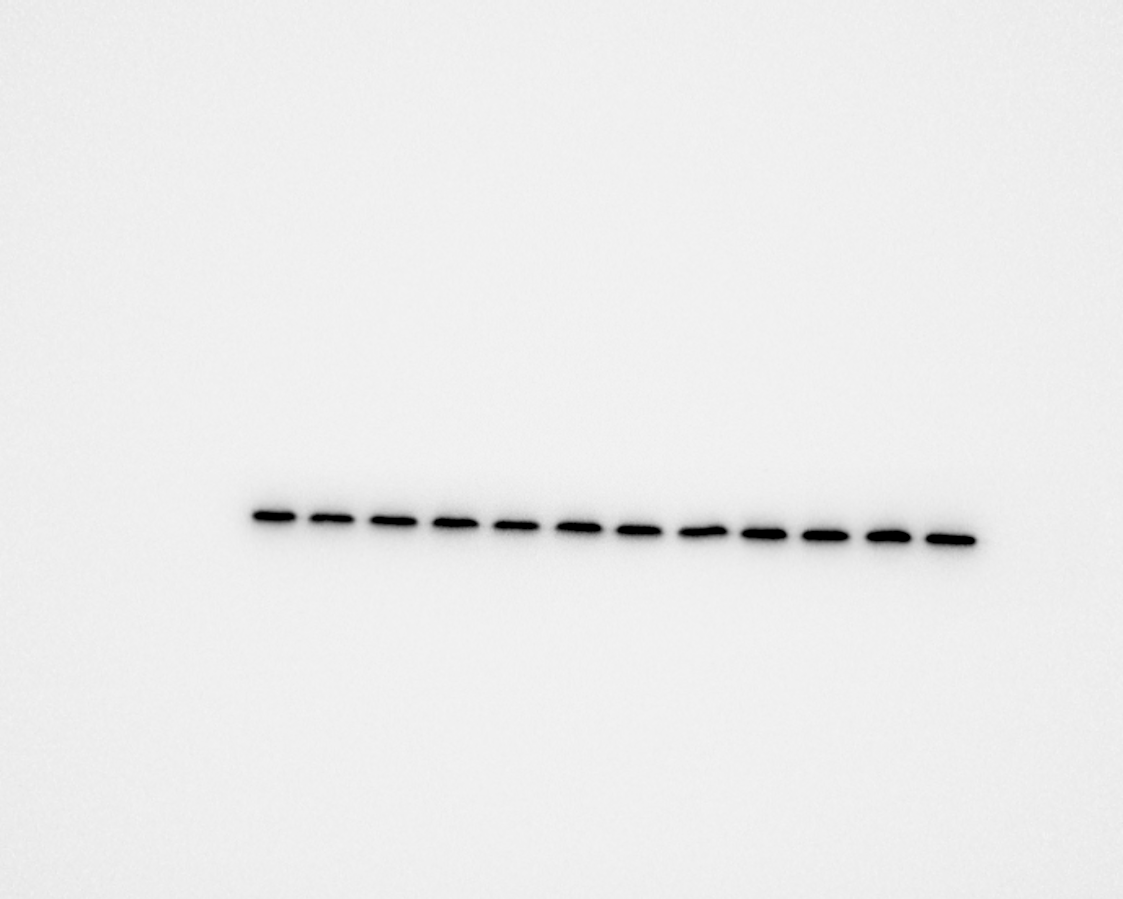

Supplement: Supplementary file 7 [file Image1.TIF]

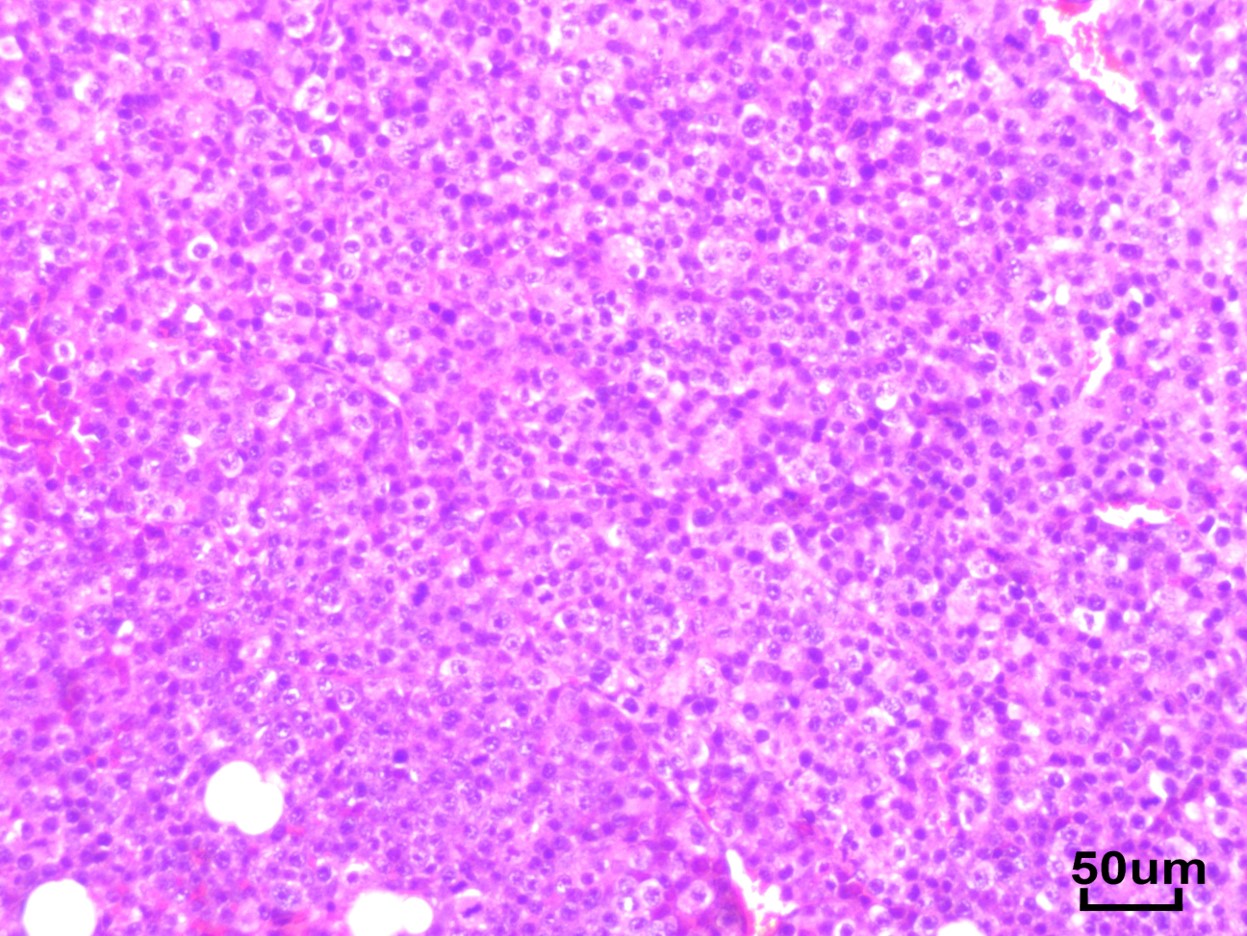

Supplement: Supplementary file 8 [file Image11.JPEG]

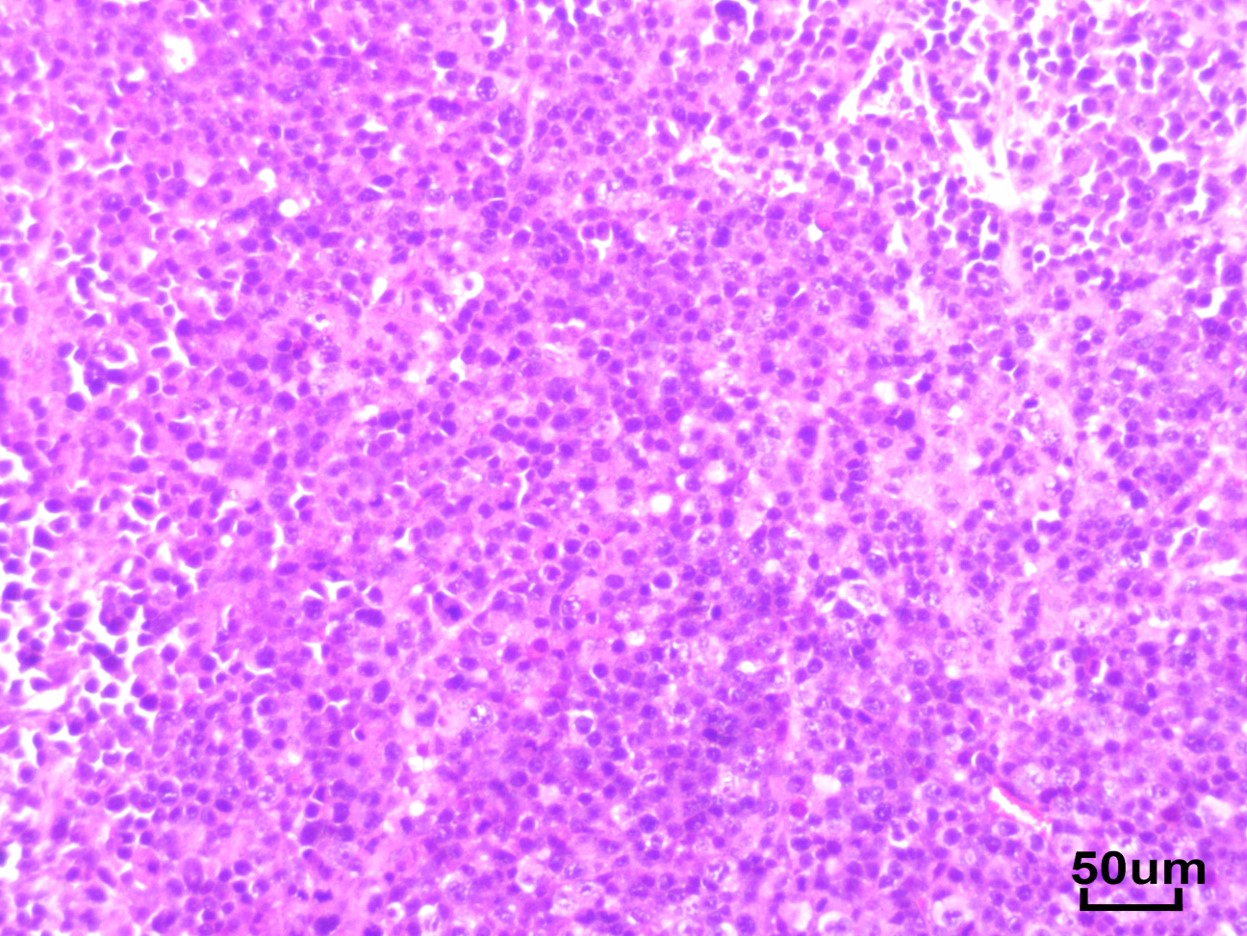

Supplement: Supplementary file 9 [file Image8.JPEG]

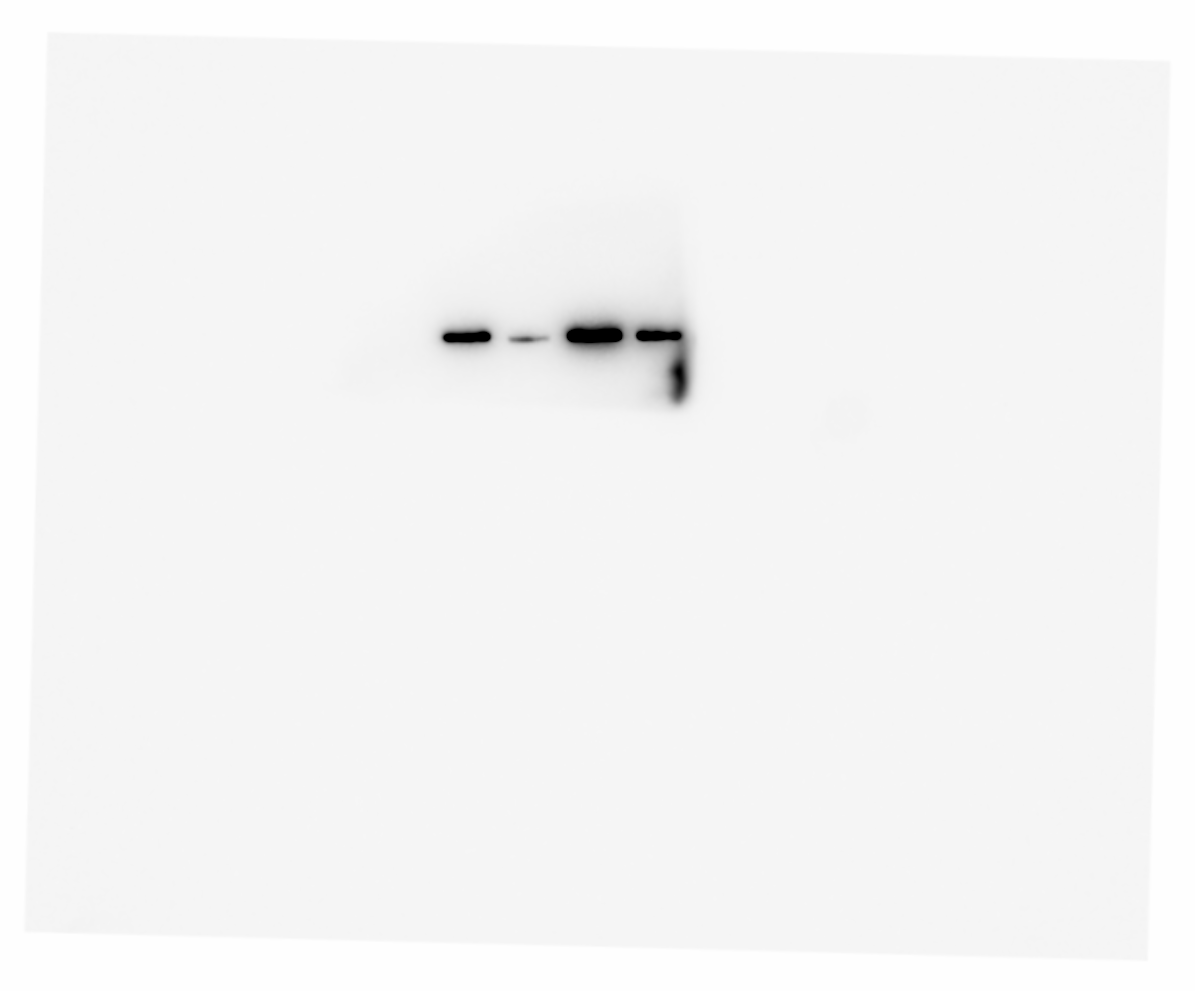

Supplement: Supplementary file 10 [file Image5.TIF]

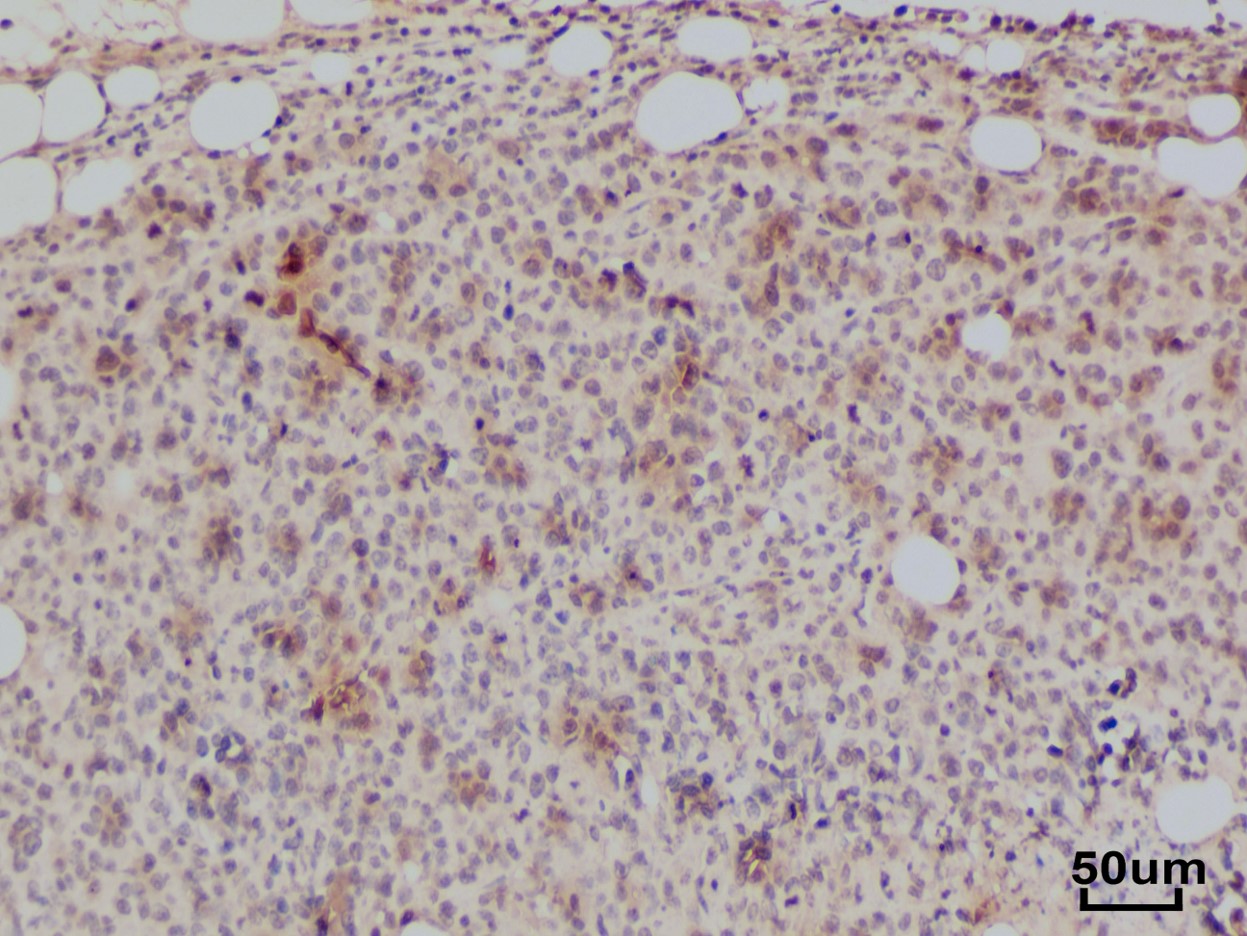

Supplement: Supplementary file 11 [file Image6.JPEG]
